# Smartphone Application‐Based Addiction Scale: Psychometric Evidence Across Nine Asian Regions Using Advanced Analytic Methods

**DOI:** 10.1002/brb3.70133

**Published:** 2024-11-17

**Authors:** I‐Hua Chen, Iqbal Pramukti, Wan Ying Gan, Kamolthip Ruckwongpatr, Le An Pham, Po‐Ching Huang, Mohammed A. Mamun, Irfan Ullah, Haitham A. Jahrami, Chung‐Ying Lin, Amir H. Pakpour

**Affiliations:** ^1^ Chinese Academy of Education Big Data Qufu Normal University Qufu China; ^2^ Department of Community Health Nursing, Faculty of Nursing Universitas Padjadjaran Sumedang West Java Indonesia; ^3^ Department of Nutrition, Faculty of Medicine and Health Sciences Universiti Putra Malaysia Serdang Selangor Malaysia; ^4^ Institute of Allied Health Sciences, College of Medicine National Cheng Kung University Tainan Taiwan; ^5^ Center of Family Medicine University of Medicine and Pharmacy at Ho Chi Minh City Ho Chi Minh City Vietnam; ^6^ School of Physical Therapy, Graduate Institute of Rehabilitation Science, College of Medicine Chang Gung University Taoyuan Taiwan; ^7^ Department of Public Health and Informatics Jahangirnagar University Dhaka Bangladesh; ^8^ CHINTA Research Bangladesh Dhaka Bangladesh; ^9^ Kabir Medical College Gandhara University Peshawar Pakistan; ^10^ Department of Psychiatry, College of Medicine and Medical Sciences Arabian Gulf University Manama Bahrain; ^11^ Psychiatric Hospital Government Hospitals Manama Bahrain; ^12^ Biostatistics Consulting Center, National Cheng Kung University Hospital, College of Medicine National Cheng Kung University Tainan Taiwan; ^13^ Department of Occupational Therapy, College of Medicine National Cheng Kung University Tainan Taiwan; ^14^ Department of Public Health, College of Medicine National Cheng Kung University Tainan Taiwan; ^15^ Department of Nursing, School of Health and Welfare Jönköping University Jönköping Sweden; ^16^ Social Determinants of Health Research Center, Research Institute for Prevention of Non‐Communicable Diseases Qazvin University of Medical Sciences Qazvin Iran

**Keywords:** problematic smartphone use, psychometrics, SABAS, smartphone addiction, smartphone dependency

## Abstract

**Introduction:**

A smartphone is a device with various functions, including *wifi*, application functions, mobile networks, ease of mobility, and the capability of using mobile data. Because of the aforementioned functions, people may use smartphones frequently. The Smartphone Application‐Based Addiction Scale (SABAS) is a six‐item questionnaire assessing smartphone addiction with promising psychometric properties. However, it is unclear if the SABAS possesses the strong psychometric properties across Asian regions. The present study aimed to examine the factor structure of the SABAS across nine Asian regions.

**Methods:**

Using datasets collected from Asian regions of Bangladesh, China, Indonesia, Iran, Malaysia, Pakistan, Taiwan, Thailand, and Vietnam, data from 10,397 participants (mean age = 22.40 years; 44.8% men) were used for analyses. All participants completed the SABAS using an online survey or paper‐and‐pencil mode.

**Results:**

Findings from confirmatory factor analysis, Rasch analysis, and network analysis all indicate a one‐factor structure for the SABAS. Moreover, the one‐factor structure of the SABAS was measurement invariant across age (21 years or less vs. above 21 years) and gender (men vs. women) in metric, scalar, and strict invariance. The one‐factor structure was invariant across regions in metric but not scalar or strict invariance.

**Conclusion:**

The present study findings showed that the SABAS possesses a one‐factor structure across nine Asian regions; however, noninvariant findings in scalar and strict levels indicate that people in the nine Asian regions may interpret the importance of each SABAS item differently. Age group and gender group comparisons are comparable because of the invariance evidence for the SABAS found in the present study. However, cautions should be made when comparing SABAS scores across Asian regions.

## Graphical abstract

10,397 participants from Bangladesh, China, Indonesia, Iran, Malaysia, Pakistan, Taiwan, Thailand, and Vietnam completed the Smartphone Application‐Based Addiction Scale (SABAS). Data from their SABAS scores support a one‐factor structure for SABAS to measure smartphone addiction.

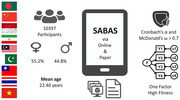

## 1 Introduction

The improvement of the smartphone functions (e.g., *wifi*, application functions, mobile networks, ease of mobility, and the capability of using mobile data) brings convenience to daily living for human beings (Tan 2023), and the prevalence of smartphone use is high worldwide. According to Turner (2024), over 85% (nearly 7 billion people) of the worldwide population had smartphones in November 2023. The benefits of smartphones have been documented for different populations, including patients who use smartphones for mHealth or eHealth programs (e.g., Callan et al. 2022; Golboni et al. 2023) and healthy people who use smartphones to engage in health behaviors (e.g., Romeo et al. 2019). However, one should not neglect the negative impacts brought by the smartphone on the health of human beings. That is, ample evidence has shown that addiction to smartphones could be harmful or associated with poor health across different populations (e.g., Chang et al. 2023; I. H. Chen et al. 2022; K. Y. Lee, Chen, et al. 2023).

Although there are no diagnostic criteria for smartphone addiction (or smartphone disorder) documented in authorized health organizations (e.g., World Health Organization and American Psychiatric Association), the term smartphone addiction has been used in the academic field with evidence (e.g., Montag, Blaszkiewicz, et al. 2015). Moreover, positive associations between smartphone addiction and health impairments have been largely acknowledged with various terminologies (e.g., C. Y. Chen et al. 2024; I. H. Chen, Pakpour, et al. 2020; Phetphum, Keeratisiroj, and Prajongjeep 2023). For example, problematic smartphone use, smartphone dependence, smartphone disorder, and excessive use of smartphones have been used in the literature (I. H. Chen, Strong, et al. 2020; Montag, Bey, et al. 2015; Montag et al. 2021), and these terms all indicate the same issue of addictive use of smartphones under the concept of generalized internet addiction (Ruckwongpatr et al. 2022). However, some differences between smartphone addiction and internet addiction have been discussed and evaluated in the literature (Alimoradi et al. 2024; Huang et al. 2024; Pakpour et al. 2023). For example, the internet may not always be used via the medium of a smartphone, while smartphone has functions other than the internet (e.g., offline games). Nevertheless, given that smartphones have *wifi* functions and many people use smartphones to connect to the internet, the present study uses consistent terms of smartphone addiction to indicate all the similar terms to avoid confusion.

Because of the positive associations found between smartphone addiction and health impairments, healthcare providers should have good instruments to screen or quickly evaluate the risk of smartphone addiction across different populations. Several instruments have been developed. For example, the Smartphone Addiction Scale (SAS) is a 33‐item questionnaire with a short version (i.e., SAS‐SV) containing 10 items originally developed in South Korea (Kwon et al. 2013). There were some limitations during the development process for the SAS, and Kwon et al. (2013) further developed the SAS‐SV with promising psychometric properties (e.g., *α* = 0.91). The Smartphone Addiction Proneness Scale (SAPS), containing 29 items, is another questionnaire originally developed in South Korea with promising psychometric properties (e.g., *α* = 0.88) (Kim et al. 2014). The Smartphone Addiction Inventory (SPAI) is a 26‐item questionnaire originally developed in Taiwan with promising psychometric properties (e.g., *α* = 0.94) (Lin et al. 2014). The Smartphone Application‐Based Addiction Scale (SABAS) is a six‐item questionnaire originally developed in Hungary with promising psychometric properties (e.g., *α* = 0.81) (Csibi, Demetrovics, and Szabo 2016; Csibi et al. 2018).

Among the instruments assessing smartphone addiction, the SABAS shares with other instruments showing robust psychometric properties (Csibi, Demetrovics, and Szabo 2016; Csibi et al. 2018). Specifically, its one‐factor structure together with other psychometric properties has been widely acknowledged and supported in different language versions, including Chinese version in China (I. H. Chen, Ahorsu, et al. 2020; Peng et al. 2023), Chinese version in Hong Kong (I. H. Chen, Strong, et al. 2020; Leung et al. 2020; Yam et al. 2019), Chinese version in Taiwan (I. H. Chen, Strong, et al. 2020; Leung et al. 2020), English version in Malaysia (Tung et al. 2022), Bahasa Indonesian version in Indonesia (Nurmala et al. 2022), Thai version in Thailand (Ruckwongpatr et al. 2024), Arabic version in the United Arab Emirates (Vally and Alowais 2022), Italian version in Italy (Soraci et al. 2021), Persian version in Iran (Lin et al. 2019), Turkish version in Turkey (Gökler and Bulut 2019), Bangla version in Bangladesh (Islam et al. 2021), Serbian version in Serbia (Vujić et al. 2023), and English version in New Zealand (Mason et al. 2022).

Apart from its strong psychometric properties, the SABAS has a theoretical background to support its usefulness in assessing smartphone addiction. That is, the SABAS is developed using the component model of addiction (Griffiths 2000, 2005), which includes the following important components defining an individual's addiction performance: salience, tolerance, mood modification, relapse, withdrawal, and conflict. Another advantage of the SABAS is its brevity: it only contains six items. Therefore, the present authors consider that further evaluation of the SABAS may benefit future healthcare providers or researchers to use the SABAS in assessing smartphone addiction. Specifically, some arguments have been made for the component model of addiction in the literature. All the psychometric evidence for the SABAS indicates that it is a one‐factor structure, as Griffiths argues that all six components are embedded in the same concept of addiction. However, another instrument based on the component model of addiction (i.e., the Bergen Social Media Addiction Scale) was found to have evidence in both one‐factor and two‐factor structures (Amendola 2023a, 2023b; Flayelle et al. 2022; Fournier et al. 2023; Fournier et al. 2024). Therefore, it is unclear if the existing evidence of one‐factor structure for SABAS is robust. Therefore, using different statistical methods (including both confirmatory and exploratory methods) to examine the factor structure of SABAS could provide strong evidence to the literature.

However, to the best of the present authors’ knowledge, all the psychometric evidence for SABAS comes from a single language version or at most two language versions (i.e., Serbian and English; Vujić et al. 2023). Subsequently, it is unclear if the one‐factor structure found for the SABAS is invariant across different language versions. Therefore, it is important to reevaluate the psychometric properties of the SABAS, especially for its factor structure, with the use of multiple datasets across different regions. Indeed, prior research has indicated the importance of having cross‐country evidence for instruments’ psychometric properties (Lecuona et al. 2024; Lin et al. 2024). In this regard, future researchers or healthcare providers could have a better understanding of the SABAS in terms of using it to assess cross‐cultural samples. The study aimed to use datasets collected from different regions in Asia (including Bangladesh, China, Indonesia, Iran, Malaysia, Pakistan, Taiwan, Thailand, and Vietnam) to apply different psychometric testing methods to understand the properties of the SABAS.

## 2 Methods

### 2.1 Data Sources and Participants

The present study used several datasets to examine the psychometric properties of the SABAS. Most of the datasets have been used for other analytical plans and published previously. Datasets from Bangladesh (Li, Chen, et al. 2023; Li, Mamun, et al. 2023; Lin et al. 2023), China (Liu et al. 2022), Indonesia (Nurmala et al. 2022; Pramukti et al. 2023), Iran (Li, Chen, et al. 2023; Li, Mamun, et al. 2023; Lin et al. 2023), Malaysia (Ghazi et al. 2023; Tung et al. 2022), Pakistan (Li, Chen, et al. 2023; Li, Mamun, et al. 2023; Lin et al. 2023), Taiwan (Liu et al. 2022), and Thailand (Yang et al. 2023) have been used for prior publication. Detailed data collection can be found in those prior publications. The entire Vietnam data and some portions of Pakistani data have not yet been used for publication.

All the data were collected using convenience sampling in the mode of online (Bangladesh, China, Indonesia, Iran, Malaysia, Pakistan, Taiwan, and Thailand) or paper‐and‐pencil (Vietnam). For online data collection, online survey platforms (e.g., *Google Form* and *SurveyMonkey*) were used via the survey link posted on social media (e.g., *Facebook* and *Instagram*) or forwarded using email/messenger. The participants were requested to read the first page introducing the study purpose and related information before they decided whether to participate or not. For those who wanted to participate, they must hit the *agree* icon before entering the survey questions. For paper‐and‐pencil mode data collection, the university faculty approached the students during their class time and informed them of the study procedure. For those students who were willing to participate, they needed to sign a written informed consent. Then, the students received the paper questionnaires from the faculty to complete.

For the present study, the only inclusion criterion was that the participant needed to have a smartphone. There were no specific exclusion criteria for the present study. Moreover, all relevant ethics committees approved the data collection for each region.

### 2.2 Measures

#### 2.2.1 Smartphone Application‐Based Addiction Scale

The SABAS contains six items asking six criteria defined by the component model of addiction (Griffiths 2000, 2005). All the items were rated using a six‐point Likert scale from *strongly disagree* (Score 1) to *strongly agree* (Score 6), with a higher score indicating a higher level of smartphone addiction (Csibi et al. 2018). The six‐item scores were summed to indicate the total score of SABAS. A sample item of the SABAS is “My smartphone is the most important thing in my life.” Moreover, prior psychometric evidence shows that the SABAS has a consistent one‐factor structure across different language versions, including Chinese, Hungarian, English, Bahasa Indonesian, Arabic, Persian, Turkish, Bangla, and Serbian (I. H. Chen, Ahorsu, et al. 2020; I. H. Chen, Strong, et al. 2020; Csibi, Demetrovics, and Szabo 2016; Csibi et al. 2018). To the best of our knowledge, no other factor structures of the SABAS have been reported in the literature. However, another instrument using the same framework of a component model of addiction (i.e., the Bergen Social Media Addiction Scale) was found to have both one‐factor and two‐factor structures in the literature (Abiddine et al. 2024; Amendola 2023a, 2023b; Fournier et al. 2023, 2024).

### 2.3 Statistical Analysis

#### 2.3.1 Descriptive Statistics

Mean and SD were used to present the central tendency of participants’ age and SABAS score (in item score and total score). Also, kurtosis and skewness values were calculated to present the distribution performance of the SABAS item scores. Frequency and percentage were used to present the descriptive statistics of the categorical data, including gender and region.

#### 2.3.2 Internal Consistency and Item‐Rest Correlation

Because the literature shows strong evidence of the one‐factor structure for the SABAS (I. H. Chen, Ahorsu, et al. 2020; I. H. Chen, Strong, et al. 2020; Csibi, Demetrovics, and Szabo 2016; Csibi et al. 2018), all six SABAS items were used to test for the internal consistency. Two forms of internal consistency were calculated: Cronbach's *α* and McDonald's *ω*, and both *α* and *ω* over 0.7 indicate acceptable internal consistency (Cheung et al. 2023; Taber 2018). Together with the internal consistency, item‐rest correlations for all the SABAS items were computed to know if the six items were mutually correlated well, and an item‐rest correlation over 0.3 indicates an acceptable correlation (Cristobal, Flavian, and Guinaliu 2007).

#### 2.3.3 Confirmatory Factor Analysis and Measurement Invariance

The confirmatory factor analysis (CFA) was used to further verify if the SABAS has a one‐factor structure and if the one‐factor structure is measurement invariant across the following variables: age groups (21 years or younger vs. above 21 years), genders (men vs. women), and regions (including Taiwan, Malaysia, Indonesia, China, Thailand, Vietnam, Bangladesh, Iran, and Pakistan). The diagonally weighted least squares (DWLS) estimator was used for the CFA. The DWLS estimator was used because we assume that the response scale in the SABAS (i.e., the six‐point Likert scale) is ordinal. The following fit indices were then used to evaluate the fit between the one‐factor structure of the present data and the one‐factor structure of SABAS: comparative fit index (CFI) larger than 0.9, Tucker–Lewis index (TLI) larger than 0.9, root mean square residual of approximation (RMSEA) < 0.08, and standardized root mean square residual (SRMR) < 0.08 (Hoyle 1995). Factor loadings of the CFA were also computed, and a factor loading larger than 0.3 is acceptable (Hair et al. 2009). Regarding the measurement invariance, multigroup CFA with four nested models was used. Specifically, the configural model, metric invariance model, scalar invariance model, and strict invariance model were constructed and compared (Cheung and Rensvold 2002; C. T. Lee, Lin, et al. 2023). When the differences were larger than −0.01 in CFI and smaller than 0.01 in RMSEA and SRMR between every two nested models, the invariance is supported (Rutkowski and Svetina 2014). A supported metric invariance indicates factor loadings were invariant across subgroups; scalar invariance indicates factor loadings and item intercepts were invariant; and strict invariance indicates factor loadings, item intercepts, and item uniqueness were invariant (Li, Mamun, et al. 2023). The CFAs were analyzed using Jeffreys' Amazing Statistics Program (JASP) version 0.18.3.0.

#### 2.3.4 Rasch Models and Differential Item Functioning

The Rasch model was conducted using the partial credit model. In addition, principal component analysis (PCA) was conducted to examine if the variance from six SABAS items only extracts one component (i.e., being unidimensional for the SABAS). When the first eigenvalue and explained variance calculated from the PCA are large and the first contrast of the eigenvalue and unexplained variance are small, the unidimensionality of the SABAS can be confirmed (Fan, Li, et al. 2023; Souza et al. 2017). Subsequently, each SABAS item was examined to see if it fits well in the unidimensional SABAS construct using the infit and outfit mean squares (MnSqs). When an item's MnSq is between 0.5 and 1.5, this item fits well in the construct (Fan, Chang, et al. 2023; Lin et al. 2023). Moreover, the Wright map was illustrated for the SABAS to portray the relationships between item difficulty and person ability (i.e., using *logits* as the *Y*‐axis unit to quantify where the items and participants should be located based on their item difficulty or person ability). The uniqueness values of the SABAS items were examined to see if they have residual correlations. The six‐point Likert scale in the SABAS was also examined to see if there were any disordered categorical functions. In addition to the item statistics, the entire SABAS and the sample were examined if they have good separation reliability and separation index: acceptable separation person/item reality should be greater than 0.7; acceptable separation person/item index should be larger than 2 (Lin et al. 2023). Finally, every SABAS item was checked if it displays substantial differential item functioning (DIF) across (21 years or younger vs. above 21 years), genders (men vs. women), or regions (including Taiwan, Malaysia, Indonesia, China, Thailand, Vietnam, Bangladesh, Iran, and Pakistan). A DIF contrast larger than 0.5 *logit* indicates substantial DIF. The Rasch analysis and DIF were analyzed using Winsteps 4.3.0.

#### 2.3.5 Network Analysis

The network analysis (NA) was conducted to explore the connections between the SABAS items and their underlying patterns. NA has been widely used to establish a model containing relationships between several variables across literature (Borsboom et al. 2021). The NA has been trying to simplify the complex relationships between psychological models. Unlike the two known statistical tests of structural equation modeling and PCA, NA considers the reciprocal relationships between the variables (Schmittmann et al. 2013). The NA was visualized using the graphical least absolute shrinkage and selection operator method based on the extended Bayesian information criterion in JASP version 0.18.3.0. In the framework of the NA, each variable is called a node (here SABAS items). The relationships (correlations) between these nodes are called edges. Several metrics are used in NA to explore the network structure, including centrality (the importance or significance of a node within a network), betweenness (the extent to which a particular node lies on the shortest paths between other nodes in the network), and closeness (how rapidly a node can interact with other nodes in the network) (Epskamp et al. 2012). The stability of the edge was assessed by estimating 95% confidence intervals through 5000 bootstrapped samples. Moreover, the NA helps illustrate the factor structure of the SABAS. The NA was analyzed using JASP version 0.18.3.0.

## 3 Results

**FIGURE 1:**
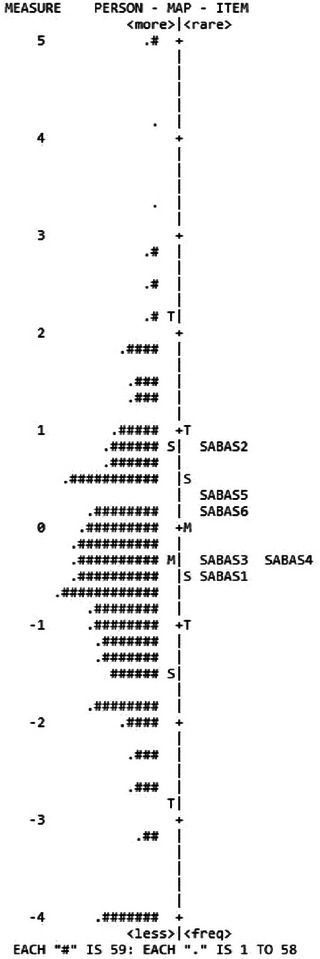
Person‐item map for the Smartphone Application‐Based Addiction Scale (SABAS) (*n* = 10,397). Each “#” is person; M, S, and T represent the mean and 1 and 2 standard deviations, respectively.

**FIGURE 2:**
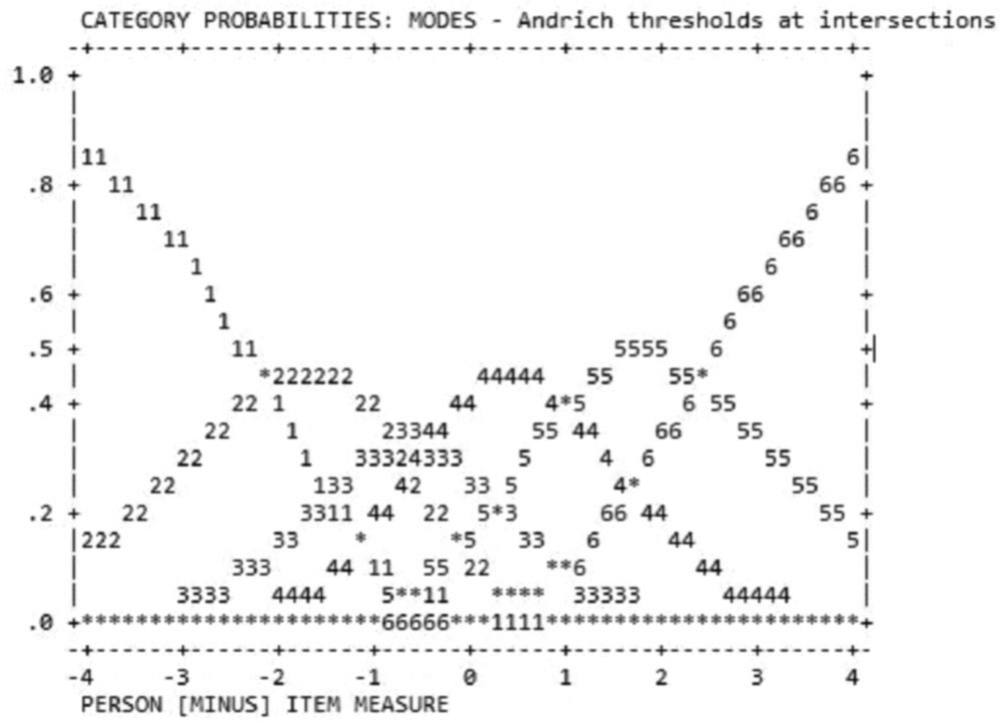
Probabilities of each response for the Smartphone Application‐Based Addiction Scale (SABAS). 1 = strongly disagree; 2 = disagree; 3 = slightly disagree; 4 = slightly agree; 5 = agree; 6 = strongly agree.

The study included 10,397 participants (mean age = 22.40 years; 44.8% men; Table 1). The item‐level statistics of the SABAS are presented in Table 2, which shows that all the items could be considered as normal distributions (absolute values of skewness and kurtosis < 1). Moreover, the items were coherent with each other (item‐rest correlations = 0.577–0.708) with good fit statistics in the Rasch model (infit MnSq = 0.84–1.32; outfit MnSq = 0.84–1.32) and factor loadings in CFA findings (value = 0.423–0.885) (Table 3). Figure 1 illustrates the Wright Map showing how each item's difficulty matches with each person's ability; Figure 2 shows that there were no disordered categorical functions in the six‐point Likert scale in the SABAS.

**TABLE 1:** Characteristics and Smartphone Application‐Based Addiction Scale (SABAS) scores across regions.

Region	*N*	Men *n* (%)	*M*_age_	SD_age_	SABAS				Language
					*M*	SD	*α*	*ω*	
Total	10,397	4659 (44.8%)	22.40	5.48	19.26	6.45	0.86	0.86	Multiple
Bangladesh	534	259 (48.5%)	22.71	4.56	20.02	6.68	0.85	0.86	Bangla
China	3135	1337 (42.6%)	19.64	2.00	19.26	6.09	0.88	0.88	Chinese^a^
Indonesia	458	119 (26.0%)	22.46	8.06	19.72	4.96	0.75	0.75	Indonesian
Iran	702	336 (47.9%)	33.29	8.64	15.37	6.21	0.84	0.84	Farsi
Malaysia	1246	928 (74.5%)	22.98	3.80	21.61	6.89	0.88	0.88	English
Pakistan	1096	460 (42.0%)	21.53	2.21	17.04	6.81	0.87	0.87	Urdu
Taiwan	1203	567 (47.1%)	26.06	6.00	21.99	5.87	0.85	0.85	Chinese^b^
Thailand	1413	433 (30.6%)	20.58	2.43	18.87	5.97	0.84	0.85	Thai
Vietnam	610	220 (36.1%)	21.09	1.74	17.40	5.40	0.81	0.81	Vietnamese

^a^ Using written language of simplified Chinese characters.

^b^ Using written language of traditional Chinese characters.

**TABLE 2:** Item‐level descriptive statistics of the Smartphone Application‐Based Addiction Scale (SABAS).

Item	Mean [range]	SD	Skewness	Kurtosis	IRC	Rasch		
						Infit MnSq	Outfit MnSq	Difficulty
1. My smartphone is the most important thing in my life.	3.63 [1, 6]	1.47	−0.25	−0.86	0.577	1.32	1.32	−0.53
2. Conflicts have arisen between me and my family (or friends) because of my smartphone use.	2.57 [1, 6]	1.34	0.56	−0.58	0.584	1.16	1.15	0.81
3. Preoccupying myself with my smartphone is a way of changing my mood (I get a buzz, or I can escape or get away, if I need to).	3.50 [1, 6]	1.71	−0.22	−0.81	0.684	0.91	0.90	−0.36
4. Over time, I fiddle around more and more with my smartphone.	3.51 [1, 6]	1.40	−0.19	−0.80	0.708	0.84	0.84	−0.38
5. If I cannot use or access my smartphone when I feel like, I feel sad, moody, or irritable.	2.97 [1, 6]	1.38	0.26	−0.78	0.688	0.88	0.89	0.30
6. If I try to cut the time I use my smartphone, I manage to do so for a while, but then I end up using it as much or more than before.	3.08 [1, 6]	1.38	0.24	−0.76	0.693	0.87	0.88	0.16

Abbreviations: Infit MnSq = information‐weighted fit mean square; IRC = item‐rest correlation; Outfit MnSq = outlier‐sensitive fit mean square.

**TABLE 3:** Fit statistics and factor loadings of the confirmatory factor analysis fitting unidimensional structure of the Smartphone Application‐Based Addiction Scale (SABAS).

Region	*χ* ^2^ (df)	CFI	TLI	RMSEA	90% CI for RMSEA	SRMR	Factor loadings
Bangladesh	9.397 (9)	1.000	1.000	0.009	0.000–0.050	0.031	0.512–0.812
China	86.162 (9)	0.993	0.988	0.052	0.043–0.063	0.042	0.592–0.825
Indonesia	12.885 (9)	0.994	0.990	0.031	0.000–0.065	0.041	0.423–0.664
Iran	15.571 (9)	0.997	0.994	0.032	0.000–0.059	0.036	0.590–0.716
Malaysia	42.492 (9)	0.993	0.988	0.055	0.039–0.072	0.044	0.661–0.799
Pakistan	17.176 (9)	0.998	0.997	0.029	0.004–0.049	0.030	0.615–0.809
Taiwan	38.422 (9)	0.991	0.985	0.052	0.036–0.070	0.046	0.556–0.804
Thailand	38.192 (9)	0.993	0.988	0.048	0.033–0.064	0.040	0.508–0.770
Vietnam	10.183 (9)	0.999	0.999	0.015	0.000–0.049	0.032	0.500–0.768

Abbreviations: CFI = comparative fit index; CI = confidence interval; RMSEA = root mean square error of approximation; SRMR = standardized root mean square residual; TLI = Tucker–Lewis index.

Regarding the factor structure of the SABAS, a unidimensional structure was confirmed in the CFA results across all the regions: CFI = 0.991–1.000; TLI = 0.985–1.000; RMSEA (90% CI) = 0.009–0.055 (0.000, 0.072); and SRMR = 0.030–0.046 (Table 3). In addition, the internal consistency findings echo the unidimensional structure (*α* = 0.75–0.88; *ω* = 0.75–0.88) (Table 1). The unidimensional structure was then agreed upon by the results of PCA in Rasch analysis (eigenvalue [variance explained] = 57.9 [82.44%] for the first component; 1.57 [11.00%] for the first contrast of the unexplained variance). Rasch model findings further indicate that the present sample and the SABAS items had good item separation reliability (value = 1.00), person separation reliability (value = 0.83), item separation index (value = 40.88), and person separation reliability (value = 2.18). No residual correlations of the SABAS item were observed in the Rasch model.

Measurement invariance was supported for the SABAS across age (21 years or less vs. above 21 years) and gender (men vs. women) in all the invariance tests (i.e., metric, scalar, and strict invariance): *∆*CFI = −0.002–0.000; *∆*SRMR = −0.002–0.003; and *∆*RMSEA = −0.004–0.001 (Table 4). Measurement invariance was supported for the SABAS across regions in metric invariance (*∆*CFI = −0.006; *∆*SRMR = 0.014; and *∆*RMSEA = 0.008) but not scalar and strict invariance (Table 4). Like the findings in measurement invariance testing, the DIF contrasts showed that all the SABAS items had no substantial DIF across gender and age. Moreover, some items displayed substantial DIF across regions (Table 5).

**TABLE 4:** Measurement invariance across age, gender, and country on the Smartphone Application‐Based Addiction Scale (SABAS) using confirmatory factor analysis.

Model and comparisons	Fit statistics							
	*χ* ^2^ (*df*)	*∆χ* ^2^ (*∆*df)	CFI	*∆*CFI	SRMR	*∆*SRMR	RMSEA	*∆*RMSEA
Age (≤ 21 years vs. > 21 years)								
M1: Configural	209.171 (18)^*^		0.994		0.034		0.045	
M2: Metric	219.845 (23)^*^		0.994		0.035		0.041	
M3: Scalar	264.960 (28)^*^		0.993		0.033		0.040	
M4: Strict	317.158 (34)^*^		0.992		0.036		0.040	
M2−M1		10.674 (5)		0.000		0.001		−0.004
M3−M2		45.115 (5)^*^		−0.001		−0.002		−0.001
M4−M3		52.198 (6)^*^		−0.001		0.003		0.000
Gender (men vs. women)								
M1: Configural	200.786 (18)^*^		0.995		0.033		0.044	
M2: Metric	233.656 (23)^*^		0.994		0.036		0.042	
M3: Scalar	290.593 (28)^*^		0.992		0.035		0.043	
M4: Strict	308.089 (34)^*^		0.992		0.036		0.039	
M2−M1		32.870 (5)^*^		−0.001		0.003		−0.002
M3−M2		56.937 (5)^*^		−0.002		−0.001		0.001
M4−M3		17.496 (6)^*^		0.000		0.001		−0.004
Region (across nine regions)								
M1: Configural	270.478 (81)^*^		0.994		0.039		0.045	
M2: Metric	514.622 (121)^*^		0.988		0.053		0.053	
M3: Scalar	2341.810 (161)^*^		0.933		0.089		0.108	
M4: Strict	2833.034 (209)^*^		0.920		0.102		0.104	
M2−M1		244.144 (40)^*^		−0.006		0.014		0.008
M3−M2		1827.188 (40)^*^		−0.055		0.036		0.055
M4−M3		491.224 (48)^*^		−0.013		0.013		−0.004

Abbreviations: CFI = comparative fit index; M1 = Model 1, a configural model; M2 = Model 2, a model based on M1 with all factor loadings constrained being equal across groups; M3 = Model 3, a model based on M2 with all item intercepts constrained being equal across groups; RMSEA = root mean square error of approximation; SRMR = standardized root mean square residual.

* *p* < 0.05.

**TABLE 5:** Differential item functioning (DIF) contrast for the Smartphone Application‐Based Addiction Scale (SABAS) in Rasch analysis across country, age, and gender.

	DIF contrast					
	Item 1	Item 2	Item 3	Item 4	Item 5	Item 6
**Region**						
1 vs. 2	−0.37	0.30	−0.07	0.32	−0.15	−0.08
1 vs. 3	0.09	0.47	0.28	**−0.64**	0.00	−0.19
1 vs. 4	−0.46	0.34	−0.21	0.16	−0.07	0.16
1 vs. 5	**−0.56**	−0.27	−0.03	0.40	−0.14	0.45
1 vs. 6	**−0.53**	−0.13	−0.23	0.45	0.26	0.03
1 vs. 7	**−1.17**	−0.07	**−0.54**	0.20	**0.82**	**0.66**
1 vs. 8	**−1.41**	−0.06	−0.04	0.07	**0.75**	**0.56**
1 vs. 9	**−1.13**	0.36	−0.07	−0.17	0.35	**0.62**
2 vs. 3	0.46	0.17	0.35	**−0.96**	0.14	−0.11
2 vs. 4	−0.10	0.04	−0.14	−0.16	0.08	0.25
2 vs. 5	−0.19	**−0.57**	0.04	0.09	0.01	**0.53**
2 vs. 6	−0.16	−0.43	−0.16	0.13	0.40	0.12
2 vs. 7	**−0.80**	−0.37	−0.47	−0.12	**0.97**	**0.75**
2 vs. 8	**−1.04**	−0.36	0.03	−0.25	**0.90**	**0.64**
2 vs. 9	**−0.76**	0.06	0.00	−0.49	0.50	**0.70**
3 vs. 4	**−0.56**	−0.13	−0.48	**0.81**	−0.06	0.36
3 vs. 5	**−0.66**	**−0.74**	−0.30	**1.05**	−0.13	**0.64**
3 vs. 6	**−0.62**	**−0.60**	−0.50	**1.09**	0.26	**0.23**
3 vs. 7	**−1.27**	**−0.54**	**−0.82**	**0.84**	**0.83**	**0.86**
3 vs. 8	**−1.50**	**−0.53**	−0.32	**0.72**	**0.75**	**0.75**
3 vs. 9	**−1.23**	−0.11	0.34	0.47	0.35	**0.82**
4 vs. 5	−0.10	**−0.61**	0.18	0.24	−0.07	0.28
4 vs. 6	−0.07	−0.47	−0.02	0.29	0.32	−0.13
4 vs. 7	**−0.71**	0.41	−0.34	0.04	**0.89**	0.50
4 vs. 8	**−0.94**	−0.40	0.16	−0.09	**0.82**	0.39
4 vs. 9	**−0.67**	0.02	0.14	−0.33	0.42	0.46
5 vs. 6	0.03	0.14	−0.20	0.04	0.39	**0.41**
5 vs. 7	**−0.61**	0.20	**−0.51**	−0.21	**0.96**	0.22
5 vs. 8	**−0.85**	0.21	−0.02	−0.33	**0.89**	**0.11**
5 vs. 9	**−0.57**	**0.63**	−0.04	**−0.58**	0.49	**0.17**
6 vs. 7	**−0.64**	0.05	−0.32	−0.25	**0.57**	**0.63**
6 vs. 8	**−0.88**	0.07	0.18	−0.38	0.49	**0.52**
6 vs. 9	**−0.60**	0.48	0.16	**−0.62**	0.09	**0.59**
7 vs. 8	−0.23	**0.01**	0.50	−0.13	−0.07	−0.11
7 vs. 9	0.04	**0.43**	0.48	−0.37	−0.47	−0.04
8 vs. 9	0.28	0.42	−0.02	−0.25	−0.40	0.06
Gender						
M vs. W	0.04	−0.18	0.11	0.02	0.00	0.00
Age						
O vs. Y	0.07	0.09	0.00	0.06	−0.18	0.00

*Note*: Region 1 = Taiwan; Region 2 = Malaysia; Region 3 = Indonesia; Region 4 = China; Region 5 = Thailand; Region 6 = Vietnam; Region 7 = Bangladesh; Region 8 = Iran; Region 9 = Pakistan; Gender: M = men; W = women; Age: O = participants with a median age of 21 years and above; Y = participants with a median age lower than 21 years old. DIF contrasts > 0.5 are **in bold**.

**FIGURE 3:**
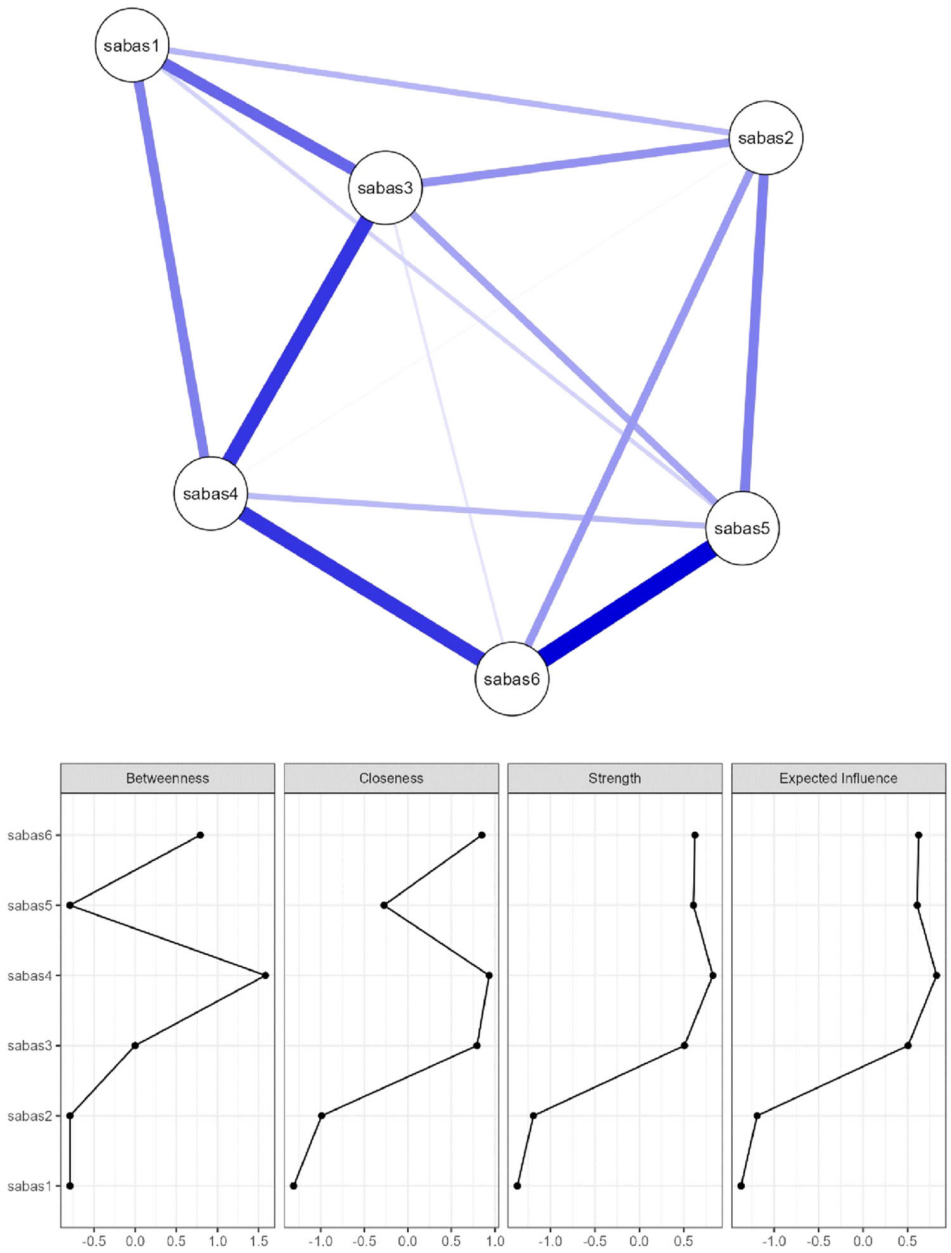
Network of Smartphone Application‐Based Addiction and centrality measures.

The NA results are presented in Figure 3, depicting a network with 15 non‐zero edges (from 15) and a sparsity of 0. As the network shows, all SABAS items (symptoms of smartphone application dependency) are interconnected with the highest edge weights between nodes SABAS Item 5 and SABAS Item 6 (*r* = 0.403). The centrality measures for all SABAS items (nodes) were above 0.5, indicating network robust connectivity. SABAS Item 4 stands out as a pivotal intermediary with the highest betweenness (1.58), closeness (0.93), and strength (0.83). The stability of the network was confirmed by the narrow‐bootstrapped 95% CI ranges of edge weights (please see the Appendix for more information).

## 4 Discussion

To the best of the present authors’ knowledge, this is the first study assessing the psychometric properties of the SABAS using data from different Asian regions, including Bangladesh, China, Indonesia, Iran, Malaysia, Pakistan, Taiwan, Thailand, and Vietnam, with relatively large sample sizes (each region contained at least 450 participants). The findings indicated that the SABAS has a one‐factor structure and this factor structure and all six items were invariant across gender and age groups. Although all the language versions of the SABAS indicate a one‐factor structure, some items were not invariant across these Asian regions. Moreover, different psychometric testing methods all indicate promising psychometric properties for the SABAS.

The present findings of the SABAS psychometric properties agree with prior studies’ findings that the SABAS has satisfactory internal consistency (e.g., I. H. Chen, Ahorsu, et al. 2020; Leung et al. 2020; Yam et al. 2019). Also, the present findings echo the prior evidence of a one‐factor structure for the SABAS in CFA (e.g., I. H. Chen, Ahorsu, et al. 2020; Leung et al. 2020; Yam et al. 2019) and the evidence of unidimensionality for the SABAS in Rasch analysis (e.g., Lin et al. 2019; Tung et al. 2022). Furthermore, the present NA findings illustrated that the six SABAS items were associated under one concept with strong correlations of items 1 with 3, items 3 with 4, items 4 with 5, and items 5 with 6; a similar pattern was found in prior studies testing the SABAS together with other measures (i.e., Barratt Impulsiveness Scale 11 and Behavioral Inhibition/Activation Systems Scales) (Guo, He, et al. 2022; Guo, Liang, et al. 2022). While NA is not traditionally used in the same way as factor analysis to test dimensionality, it can still provide valuable insights into the structure of a scale by showing high intercorrelations, and clustering of items within a network can indicate that all items may be measuring the same underlying construct (Borsboom and Cramer 2013). Therefore, the unidimensionality of the SABAS is fully supported by the present study's findings that agreed with the previous psychometric evidence on SABAS.

All the present psychometric testing findings indicate that the SABAS has a one‐factor structure, which can be explained by the unidimensional concept of the component model of addiction proposed by (Griffiths 2000, 2005). Indeed, the six components mentioned in the component model of addiction are all centered on the concept of “addiction.” That is, only when individuals with the issue of addiction would have problems with salience, tolerance, mood modification, relapse, withdrawal, and conflict. Although some evidence in the literature shows that the component model of addiction does not distinguish peripheral (i.e., salience, tolerance) and central features of addiction for behavioral addictions (Fournier et al. 2023), the component model of addiction contains the basis of a confirmatory approach to classify behavioral addiction (Griffiths 2019; Kim and Hodgins 2018). Therefore, we considered that the findings of the one‐factor structure for the SABAS fit well with the confirmatory approach in the behavioral addiction. However, the confirmatory approach has been criticized for simplifying the complex and multi‐determined phenomena in non‐substance‐related addictive behaviors (Flayelle et al. 2022), and additional evidence is warranted to corroborate the one‐factor structure for the SABAS.

Our findings indicated that the use of SABAS could be invariant across gender and age but not country. This implies that different genders and different age groups interpret the SABAS in similar ways (i.e., the different groups will consider the importance of every SABAS item in a similar sequence). However, the measurement invariance of SABAS was not supported to be across regions. This may be because different regions may include large differences in interpreting the “threshold” of smartphone use (i.e., how much is too much may be different across regional cultures). For example, prior evidence shows that greater compulsive usage of smartphones was related to greater collectivistic attitudes (Nolin 2015). In other words, people living in a society that appreciates more collectivism may have a higher threshold to define smartphone addiction. However, the present study did not collect any measures on cultural aspects; therefore, future studies are needed to clarify what factors in the cultural aspects impact people's interpretations of smartphone addiction.

There are some limitations in the present study. First, the present study used a secondary dataset, and there might be some time differences between the periods of data collection across these regions. Specifically, data in some regions was collected earlier and some were collected later. Therefore, considering the technology changes over time, the feelings toward smartphone use might be somewhat different between these collection periods. Second, only one psychometric instrument (i.e., SABAS) was analyzed in the present study. Therefore, the lack of external criterion measures could not provide other important validity information (e.g., concurrent validity) for the SABAS in the present study. Although some previous studies using the regional data have reported concurrent validity^1^, the measures were somewhat different across these studies. Third, following the previous limitation, no gold standard was used in the present study, and thus criterion‐related validity could not be examined and presented in the present study. Fourth, no other Asian regions’ data were included in the present study; therefore, the present findings might not be generalized to other Asian regions. The data were collected using convenience sampling; therefore, the present findings might have restricted generalizability.

## 5 Conclusion

In conclusion, the present study findings showed that the SABAS possesses a one‐factor structure across nine Asian regions. The one‐factor structure was supported by different psychometric testing methods, including CFA, Rasch analysis, and NA. Measurement invariance and DIF testing indicated that the SABAS entire scale and its six items were invariant across genders and age groups; therefore, comparing SABAS across genders and age is appropriate. However, cautions should be made when using SABAS to compare levels of smartphone addiction across these Asian regions because the importance of each SABAS item may not be in the same order across these Asian regions. Nevertheless, evidence showing the one‐factor structure of the SABAS in the present study could help healthcare providers to use the SABAS to assess an overall level of smartphone addiction for people across the nine regions, regardless of their age or gender.

## Author Contributions

**I‐Hua Chen**: writing–original draft, investigation; methodology, conceptualization. **Iqbal Pramukti**: methodology, investigation, writing–original draft, conceptualization. **Wan Ying Gan**: methodology, investigation, conceptualization. **Kamolthip Ruckwongpatr**: conceptualization, investigation, methodology. **Pham Le**: conceptualization, investigation, methodology. **Po‐Ching Huang**: conceptualization, investigation, methodology. **Mohammed A. Mamun**: conceptualization, investigation, methodology. **Irfan Ullah**: conceptualization, investigation, methodology. **Haitham A. Jahrami**: conceptualization, methodology, writing–original draft. **Chung‐Ying Lin**: conceptualization, investigation, writing–original draft, methodology, writing–review and editing, supervision. **Amir H Pakpour**: conceptualization, investigation, methodology, validation, supervision, formal analysis, writing–review and editing, writing–original draft.

## Ethics Statement

The study protocol is approved by the Institute of Allergy and Clinical Immunology of Bangladesh; the Human Experimental Ethics Committee in Guangzhou Sport University, China; the Health Research Ethics Commission, Faculty of Nursing, Universitas Airlangga, Indonesia; the Qazvin University of Medical Sciences, Iran; the ethics committee of Universiti Putra Malaysia, Malaysia; the Department of Psychology, University of Sargodha, Pakistan; the Institutional Review Board of Chi Mei Medical Center, Taiwan; the Human Research Ethics of National Cheng Kung University in conjunction with the ethics committee of Mahidol University, Thailand and the Board of Ethics in Biomedical Research at University of Medicine and Pharmacy at Ho Chi Minh City, Vietnam.

## Conflicts of Interest

The authors declare no conflicts of interest.

### Peer Review

The peer review history for this article is available at https://publons.com/publon/10.1002/brb3.70133

## Figures and Tables

**FIGURE A1:**
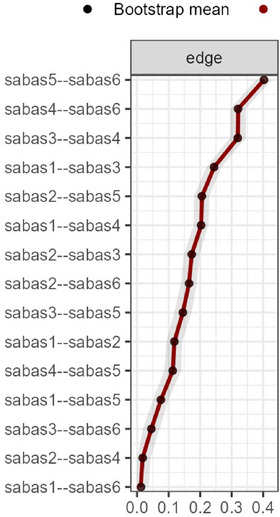
Edge stability.

**FIGURE A2:**
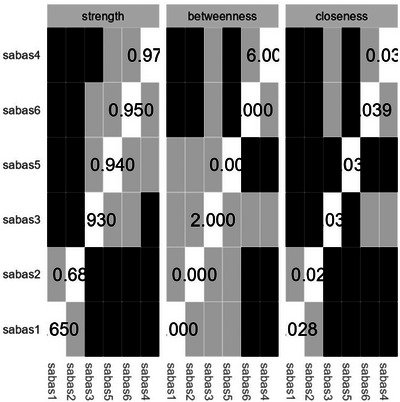
Central stability.

## Data Availability

The data that support the findings of this study are available from the corresponding author upon reasonable request.

## Endnotes

1 Data from Bangladesh, Iran, and Pakistan (Li, Chen, et al. 2023; Li, Mamun, et al., 2023; Lin et al. 2023) included the following measures in addition to SABAS: Nomophobia Questionnaire, Internet Disorder Scale–Short Form, and Phubbing Scale. Data from China and Taiwan (Liu et al. 2022) included the following measures in addition to SABAS: Bergen Social Media Addiction Scale, Nomophobia Questionnaire, Weight Self‐Stigma Questionnaire, and International Physical Activity Questionnaire Short Form. Data from Indonesia (Nurmala et al. 2022; Pramukti et al. 2023) included the following measures in addition to SABAS: Bergen Social Media Addiction Scale, Nomophobia Questionnaire, and Depression, Anxiety, Stress Scale‐21. Malaysia data (Tung et al. 2022; Ghazi et al. 2023) included the following measures in addition to SABAS: Bergen Social Media Addiction Scale, Internet Gaming Disorder Scale‐Short Form, Gaming Disorder Test, Gaming Disorder Scale for Young Adults, and Nomophobia Questionnaire. Thailand data (Yang et al. 2023) included the following measures in addition to SABAS: Assessment of Criteria for Specific Internet‐Use Disorders and Internet Gaming Disorder Scale–Short Form.

## Appendix

Figure A1

Figure A2
